# PDGF-BB regulates the pulmonary vascular tone: impact of prostaglandins, calcium, MAPK- and PI3K/AKT/mTOR signalling and actin polymerisation in pulmonary veins of guinea pigs

**DOI:** 10.1186/s12931-018-0829-5

**Published:** 2018-06-19

**Authors:** Annette D. Rieg, Said Suleiman, Carolin Anker, Eva Verjans, Rolf Rossaint, Stefan Uhlig, Christian Martin

**Affiliations:** 1 Department of Anaesthesiology, Medical Faculty RWTH-Aachen, Aachen, Germany; 2 Institute of Pharmacology and Toxicology, Medical Faculty RWTH-Aachen, Aachen, Germany

## Abstract

**Background:**

Platelet-derived growth factor (PDGF)-BB and its receptor PDGFR are highly expressed in pulmonary hypertension (PH) and mediate proliferation. Recently, we showed that PDGF-BB contracts pulmonary veins (PVs) and that this contraction is prevented by inhibition of PDGFR-β (imatinib/SU6668). Here, we studied PDGF-BB-induced contraction and downstream-signalling in isolated perfused lungs (IPL) and precision-cut lung slices (PCLS) of guinea pigs (GPs).

**Methods:**

In IPLs, PDGF-BB was perfused after or without pre-treatment with imatinib (perfused/nebulised), the effects on the pulmonary arterial pressure (P_PA_), the left atrial pressure (P_LA_) and the capillary pressure (P_cap_) were studied and the precapillary (R_pre_) and postcapillary resistance (R_post_) were calculated. Perfusate samples were analysed (ELISA) to detect the PDGF-BB-induced release of prostaglandin metabolites (TXA_2_/PGI_2_). In PCLS, the contractile effect of PDGF-BB was evaluated in pulmonary arteries (PAs) and PVs. In PVs, PDGF-BB-induced contraction was studied after inhibition of PDGFR-α/β, L-Type Ca^2+^-channels, ROCK/PKC, prostaglandin receptors, MAP2K, p38-MAPK, PI3K-α/γ, AKT/PKB, actin polymerisation, adenyl cyclase and NO. Changes of the vascular tone were measured by videomicroscopy. In PVs, intracellular cAMP was measured by ELISA.

**Results:**

In IPLs, PDGF-BB increased P_PA_, P_cap_ and R_post_. In contrast, PDGF-BB had no effect if lungs were pre-treated with imatinib (perfused/nebulised). In PCLS, PDGF-BB significantly contracted PVs/PAs which was blocked by the PDGFR-β antagonist SU6668. In PVs, inhibition of actin polymerisation and inhibition of L-Type Ca^2+^-channels reduced PDGF-BB-induced contraction, whereas inhibition of ROCK/PKC had no effect. Blocking of EP_1/3_- and TP-receptors or inhibition of MAP2K-, p38-MAPK-, PI3K-α/γ- and AKT/PKB-signalling prevented PDGF-BB-induced contraction, whereas inhibition of EP_4_ only slightly reduced it. Accordingly, PDGF-BB increased TXA_2_ in the perfusate, whereas PGI_2_ was increased in all groups after 120 min and inhibition of IP-receptors did not enhance PDGF-BB-induced contraction. Moreover, PDGF-BB increased cAMP in PVs and inhibition of adenyl cyclase enhanced PDGF-BB-induced contraction, whereas inhibition of NO-formation only slightly increased it.

**Conclusions:**

PDGF-BB/PDGFR regulates the pulmonary vascular tone by the generation of prostaglandins, the increase of calcium, the activation of MAPK- or PI3K/AKT/mTOR signalling and actin remodelling. More insights in PDGF-BB downstream-signalling may contribute to develop new therapeutics for PH.

## Background

Regulation of platelet-derived growth factor (PDGF)-BB and its receptor PDGFR-β are strongly involved in the pathogenesis of pulmonary hypertension (PH) [1, 2], as they highly act proliferative on pulmonary vessel [3]. This instance provides for the fact that PDGFR-inhibition by tyrosine kinase inhibitors (TKIs), e.g. imatinib, resembles a new intriguing approach to treat PH, as it counteracts the vascular remodelling [4]. Recent research also revealed considerable pulmonary vasorelaxant effects of TKIs, e.g. imatinib relaxes the pulmonary arterial bed of healthy and pulmonary hypertensive rats [5, 6]. Within this context, the relaxant effects of TKIs appear to be not limited to the pulmonary arterial bed, as imatinib, just as the PDGFR-β-inhibitors SU6668 or DMPQ also relax pulmonary veins (PVs) [7]. With regard to imatinib, it even exerts pulmonary venous relaxation if it is inhaled [7]. The dual action of imatinib on pulmonary vascular remodelling and vessel tone [2, 5–7] is still more remarkable, as PDGF-BB also contracts PVs [7]. Consecutively, aside the involvement in vascular remodelling [2, 3], PDGF-BB and PDGFR appear to regulate the tone of pulmonary vessels. In this regard, previous studies in systemic vessel revealed conflictive results of PDGF, e.g. contraction of the basilar artery [8] or aorta [9, 10], but relaxation of the mesenteric artery [11, 12].

PDGFR consists of two subunits, either αα, αβ or ββ and all of them are assigned to various functions, e.g. PDGFR-α is involved in organogenesis (lungs, skin, gonads or central nervous system), whereas PDGFR-β is responsible for the formation of vessel [3] and for proliferation in pulmonary vascular remodelling [1]. The various PDGFR subunits are activated by different ligands, e.g. in vivo PDGFR-α is activated by PDGF-AA or PDGF-CC, whereas PDGFR-β is activated by PDGF-BB [3]. In contrast, more possibilities are conceivable in vitro, e.g. the activation of PDGFR-αβ by PDGF-BB [3].

We designed this study to evaluate the contractile effects of PDGF-BB on the pulmonary arterial and venous bed in isolated perfused lungs (IPL) of guinea pigs (GPs) [7, 13, 14]. Further, we analysed the PDGF-BB-induced release of the prostaglandins TXA_2_ and PGI_2_ in supernatants of IPL-perfusate samples. Next, we compared the contractile effect of PDGF-BB in pulmonary arteries (PAs) or PVs after or without inhibtion of PDGFR-α (ponatinib) or PDGFR-β (SU6668) in GPs’ precision-cut lung slices (PCLS) [13, 15, 16]. Further, we studied the mechanisms beyond PDGF-BB-induced contraction in PVs. In this context, we examined the involvement of L-Type Ca^2+^-channels, Ca^2+^-sensitisation (ROCK/PKC), prostaglandin receptors and cellular pathways such as p38-MAPK, MAP2K, PI3K-α/γ, or AKT/PKB. Beyond that, we evaluated the impact of signalling cascades generally attributed to vasorelaxation; e.g. PGI_2_, cAMP or NO. Within the framework of the above mentioned signalling cascades, smooth muscle cell (SMC) contraction depends on myosin light chain (MLC) phosphorylation, regulated either by Ca^2+^-sensitisation or by the increase of intracellular calcium [17–25]. Aside MLC phosphorylation, SMC contraction depends on actin polymerisation and cytoskeletal remodeling [26, 27] which we inhibited by cytochalasin D and latrunculin A.

PCLS resembles an ex vivo model which allows to study the tone of PAs, PVs and airways concurrently within their tissue organisation excluding the exposure to in vivo factors such as shear stress, vascular filling pressure or thromboembolism [13, 15, 16, 28]. As a major advantage, PCLS allow to compare how pulmonary vessel or airways react to several stimulants within the different species [13, 28–30].

With regard to PH, there are multiple open questions concerning the role of PDGF-BB and PDGFR. We adressed the following points: 1) Does PDGF-BB contract in addition to PVs also PAs and is this contraction related to PDGFR-β? 2) How does PDGF-BB alter P_PA_, P_cap_, R_pre_ and R_post_ in IPLs? 3) How does PDGF-BB affect the pulmonary vascular tone, if lungs are pre-treated with the TKI imatinib (perfused/inhaled)? 4) What are the mechanisms beyond PDGF-BB-induced contraction?

## Methods

### Lung tissue from GPs’

Female Dunkin Hartley GPs (350 ± 50 g) were delivered from Charles River (Sulzfeld, Germany). All animal experiments were approved by the Landesamt für Natur, Umwelt und Verbraucherschutz Nordrhein-Westfalen (ID: 84–02.04.2013A146, 8.87–51.05.20.10.245 and 50066A4) and strictly performed according to the rules of the Directive 2010/63/EU of the European Parliament.

### Isolated perfused lungs of the GP

GPs’ lungs were prepared as described [7, 13, 14]. In brief, intraperitoneal anaesthesia was performed (pentobarbital: 95 mg kg^− 1^) and verified by missing reflexes. The animal was exsanguinated, the trachea cannulated and the lung ventilated with positive pressure (70 breaths/min). The apex of the left ventricle was cut and cannulas were placed in the PA (perfusion inflow) and in the left atrium (perfusion outflow). The lung was perfused at constant flow (20 mL/min) with Krebs-Henseleit buffer, containing 2% bovine serum albumin, 0.1% glucose, 0.3% HEPES and 50 nM salbutamol to prevent bronchoconstriction [31]. The temperature of the perfusate was maintained at 37 °C with a water bath and the pH was adjusted between 7.35 and 7.45 by gassing with CO_2_. Heart and lungs were withdrawn and transferred into a negative-pressure chamber, the so-called artificial thorax chamber. Next, ventilation was switched from positive pressure to negative pressure. To prevent the formation of lung oedema during constant flow perfusion and negative pressure ventilation, a pressure balancing chamber was established in the perfusion outflow which was connected by tubing to the artificial thorax chamber. To prevent atelectasis of the lung, every 5 min a deep breath was applied. Tidal volume (TV), dynamic compliance (Cdyn), resistance (Res), pulmonal arterial pressure (P_PA_), left atrial pressure (P_LA_) and the flow were continuously monitored. Further, the capillary pressure (P_cap_) was measured every 10 min by the double occlusion method [14] and the precapillary (R_pre_) and postcapillary resistance (R_post_) were calculated by the following equations: R_pre_ = $\frac{PPA- Pcap}{flow}$ and R_post_ = $\frac{Pcap- PLA}{flow}$.

**Fig. 1:**
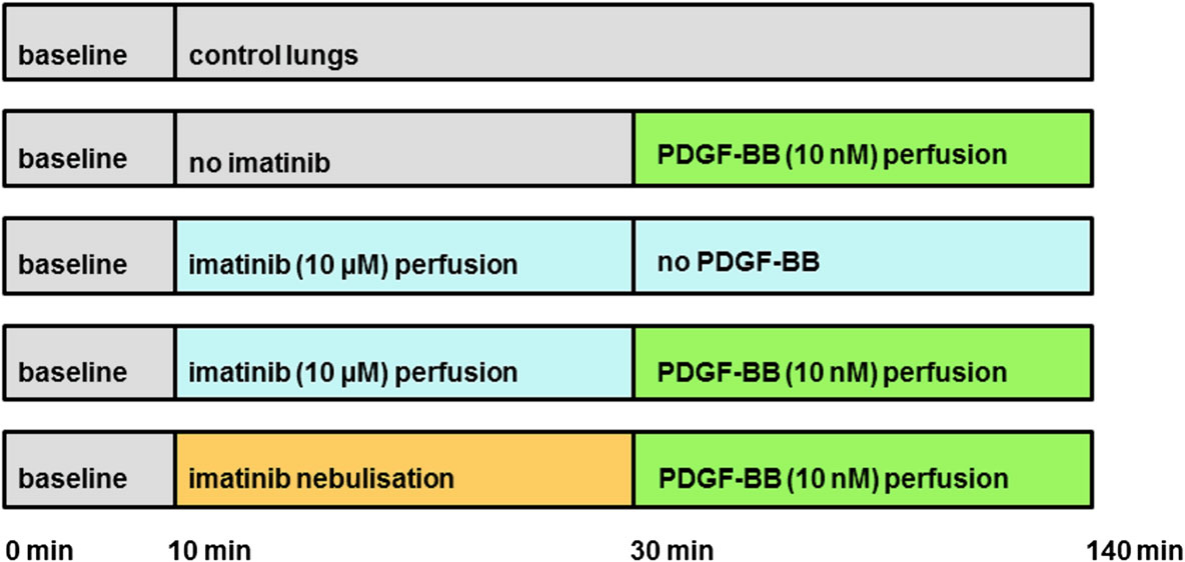
Overview of the timeline. This overview illustrates the different groups and the timeline of all experiments using the IPL

As soon as respiratory and haemodynamic parameters remained stable over 10 min (baseline), imatinib (10 μM) was perfused at time point 10 min. At a buffer volume of 200 mL, a concentration of 10 μM imatinib corresponds to a total dose of 1.18 mg imatinib or to 3.5 mg/kg body weight imatinib, respectively. Control lungs remained untreated. Next, PDGF-BB (10 nM) was added to the recirculating perfusion buffer (total volume 200 mL) at time point 30 min and perfused in untreated lungs and imatinib-pre-treated lungs. Beyond that, imatinib mesylate was nebulised in some lungs prior to the perfusion of PDGF-BB. Therefore, 29.38 mg imatinib mesylate were solved in 3 ml aqua to obtain a solution of 16.6 mM and nebulised over a period of 130 min. Assuming a lung flow of 0,21 L/min (70 breaths à 3 mL) and a pressure of 1.5 bar, the total amount of inhaled imatinib corresponds to less than 4% of the nebulised amount of imatinib [32], namely 1.18 mg, corresponding to 3.5 mg/kg body weight imatinib, respectively. To measure PGI_2_ and TXA_2_, IPL-perfusate samples were obtained at time point 0, 30 (before the application of PDGF-BB) and 120 min. The different groups and the timeline of the experiments are illustrated in Fig. 1.

### Precision-cut lung slices (PCLS) from GPs

In GPs, intraperitoneal anaesthesia was performed with 95 mg kg^− 1^ pentobarbital (Narcoren; Garbsen, Germany) and verified by missing reflexes. The GP was bled, the trachea cannulated and the diaphragm opened. Thereafter, PCLS were prepared as described before [13, 16, 30]. Whole lungs were filled via the trachea with 1.5% low-melting agarose and cooled on ice to harden the lungs. Afterwards, tissue cores (diameter 11 mm) were prepared and cut into 300 μm thick slices with a Krumdieck tissue slicer (Alabama Research & Development, Munford, AL, USA). PCLS were incubated at 37 °C and repeated medium changes were performed to wash out the agarose.

### Identification of the vessels, histology

Pulmonary vessels from GPs were identified by their anatomical landmarks; e.g. PAs accompany the airways and PVs lie aside [13, 16].

### Pharmacological interventions, measurements and videomicroscopy

To evaluate the contractile effect of PDGF-BB in PAs/PVs from GPs, PCLS were exposed for 60 min to 100 nM PDGF-BB (Figs. 3, 4 and 5, Figs 7, 8 and 9). If a signalling pathway was evaluated (Figs. 3,4 and 5, Figs 7, 8 and 9), PCLS were additionally pre-treated for 1 h with one of the following inhibitors at concentrations about 10–100 fold above the IC_50_ value of the target: PDGFR-α: 100 nM ponatinib (IC_50_: 1.1 nM) [33–35]; PDGFR-β: 5 μM SU6668 (IC_50_: 0.008–0.1 μM) [36–38]; PDGFR-α/β: 100 μM imatinib (IC_50_: 0.6–1.8 μM) [39]; L-Type Ca^2+^-channels: 100 nM amlodipine (IC_50_: 1.9 nM) [40]; Rho-Kinase: 10 μM fasudile (IC_50_: 1.4 μM) [41]; protein kinase C (PKC): 5 μM calphostin C (IC_50_: 50 nM) [42]; cyclooxygenase 1/2: 3 μM indomethacin (IC_50_: 13–26 nM) [43, 44]; EP_1_: 1 μM SC51322 (IC_50_: 13.8 nM) [45]; EP_2_: 1 μM PF04418948 (IC_50_: 2.7 nM) [46, 47]; EP_3_: 1 μM L798106 (IC_50_: 10 nM) [48, 49]; EP_4_: 1 μM L161982 (IC_50_: 3.2 nM) [48]; TP: 10 μM SQ29548 (IC_50_ 10 nM) [48]; IP: 1 μM RO-1138452 (IC_50_: 5–10 nM) [50]; MAP2K: 50 μM PD98059 (IC_50_: 2–7 μM) [51]; MAP2K: 5 μM U0126 (IC_50_: 58–72 nM) [52]; p38-MAPK: 5 μM SB203580 (IC_50_: 0.5 μM, for AKT/PKB 3–5 μM) [53, 54]; PI3K-α: 100 nM GSK 1059625 (IC_50_: 2 nM); PI3K-γ: 100 nM AS252424 (IC_50_: 33 nM) [55]; AKT/PKB: 10 μM 10-DEBC (IC_50_: 2 μM) [56]; actin polymerisation: 10 μM cytochalasin D (IC_50_: 100 nM) [57] or 1 μM latrunculin A [58]; adenyl cyclase (AC): 100 μM SQ22536 (IC_50_: 1.4–200 μM) [59] and NO-synthase (NOS): 100 μM L-NAME (IC_50_: 25 μM).

In PCLS, all changes of the initial vessel area (IVA) were quantified in % and indicated as “Change [% of IVA]”. Thus, an IVA < 100% indicates contraction and an IVA > 100% indicates relaxation. To compare the contractile effect of PDGF-BB in pre-treated vessels, the intraluminal area was defined after pre-treatment again as 100%. In the graphs, all pre-treatments were indicated. The intraluminal area of PAs and PVs was monitored with a digital video camera (Leica Viscam 1280, Leica DFC 280). The images were analysed with Optimas 6.5 (Media Cybernetics, Bothell, WA).

### ELISAs

To analyse cAMP, PVs were isolated out of tissue cores guided by their anatomical landmarks, e.g. the PAs accompany the airways and PV lies aside. PVs were incubated in medium, flushed with PDGF-BB (100 nM) and after 30 min frozen by liquid nitrogen. Cyclic AMP was quantified with ELISA-kits following the manufacturer’s protocol. Samples/standards were acetylated for stabilisation. To measure cAMP, all samples were diluted 1:2 with 0.1 M HCL. The ELISA was analysed at 405 nM (GENIOS, Tecan, Switzerland).

To analyse prostacyclin (synonym: prostaglandine I_2_ (PGI_2_)) and thromboxane A_2_ (TXA_2_), IPL perfusate samples were obtained at 0, 30 (before PDGF-BB was applied) or 120 min and stored at − 80 °C. PGI_2_ and TXA_2_ are quickly metabolised, hence the metabolites 6-keto prostaglandin F_1α_ (6-keto PGF_1α_) and 11-dehydro TXB_2_ and 2,3-dinor (TXB_2_) were measured to estimate the generation of PGI_2_ and TXA_2_, respectively. Prostaglandin metabolites were quantified with ELISA-kits following the manufacturer’s protocol and measured at 412 nM (GENIOS, Tecan, Switzerland).

### Chemicals

PDGF-BB was provided by Peprotech (Hamburg, Germany). Imatinib mesylate, amlodipine, fasudile, calphostin C, indomethacin, SC51322, PF04418948, L798106, L161982, GSK 1059615, AS 252424, 10-DEBC and SQ22536 were purchased from Tocris Bioscience (Ellisville, Missouri, USA). Ponatinib was acquired from SelleckChem (Munich, Germany). SQ29548, RO-1138452, SU6668, SB203580, PD98059 and U0126 were acquired from Cayman Europe (via Biomol, Hamburg, Germany). The cAMP ELISA-kit was acquired from Enzo (Lörrach, Germany), whereas all ELISA-kits applied to quantify prostaglandin generation were acquired from Cayman Europe (via Biomol, Hamburg, Germany). L-Name, cytochalasin D, latrunculin A or standard laboratory chemicals were provided by Sigma (Steinheim, Germany).

### Statistical analysis

Statistics were conducted using SAS software 9.3 (SAS Institute, Cary, North Carolina, USA) and GraphPad Prism 5.01 (GraphPad, La Jolla, USA). The data in Fig. 6a/c were analysed by the Wilcoxon signed rank test (matched pairs), whereas the data in Fig. 6b/dor Fig. 8c were analysed by the Mann-Whitney U test (no matched paired). All other data were analysed using a linear mixed model analysis (LMM) with the covariance structure AR(1). All *p*-values were adjusted for multiple comparisons by the false discovery rate and are presented as mean ± SEM; n indicates the numbers of animals. *P* < 0.05 was considered as significant.

## Results

We studied the pulmonary vascular effects of PDGF-BB using healthy lungs (IPL/PCLS) from GPs. Beyond that, we studied the downstream-signalling of PDGF-BB-induced contraction in PVs of GPs.

### IPL: Effect of PDGF-BB on the pulmonary vascular tone

**Fig. 2:**
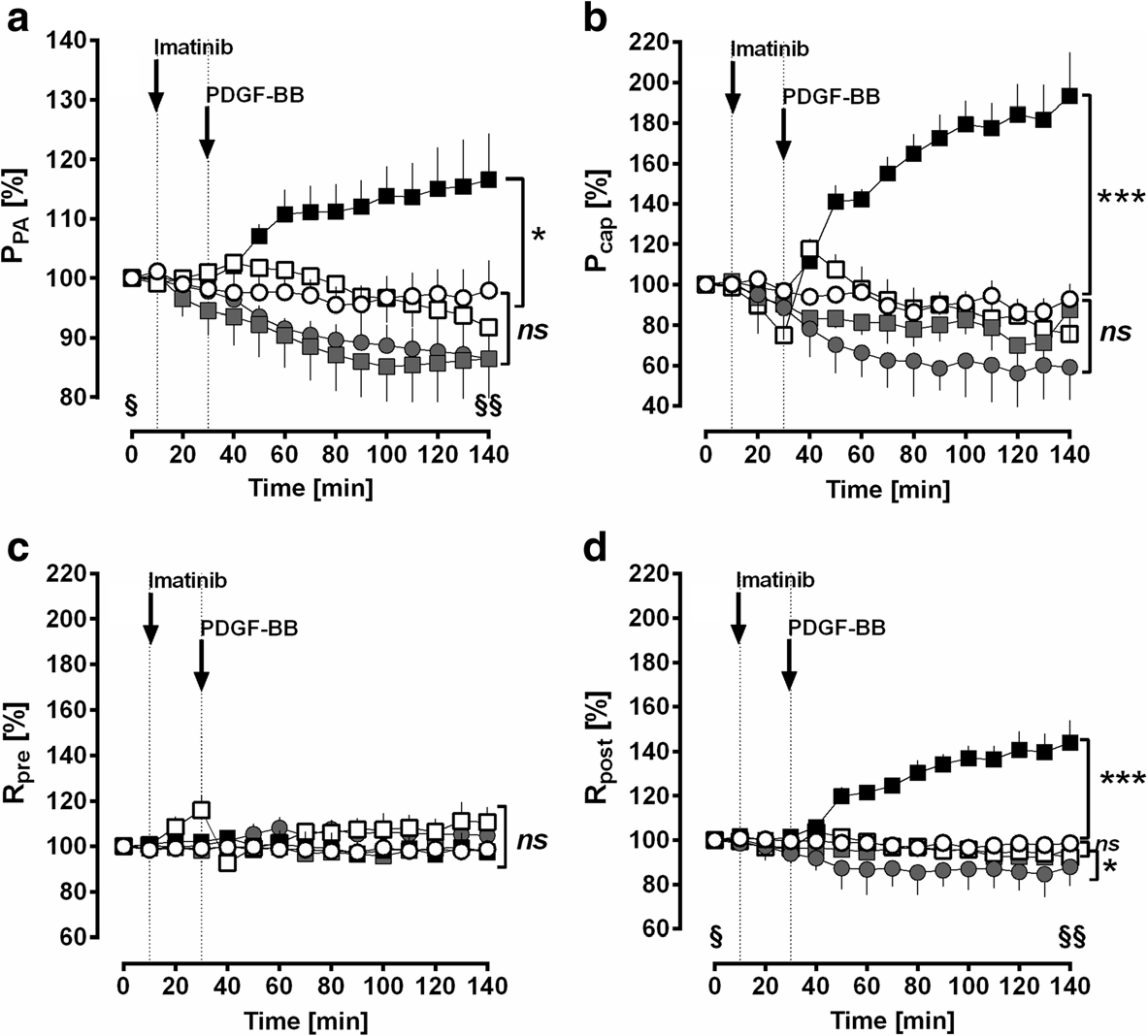
IPL: Effect of PDGF-BB on the pulmonary vascular tone. **a** Effect of PDGF-BB on P_PA_. **b** Effect of PDGF-BB on P_cap._ **c** Effect of PDGF-BB on R_pre_. **d** Effect of PDGF-BB on R_post_. For all: (○) control (*n* = 7); (■) PDGF-BB (n = 7); (grey circle) imatinib (*n* = 7); (grey square) perfused imatinib / PDGF-BB (*n* = 7); (□) nebulised imatinib / PDGF-BB (*n* = 6); **a-d** Statistics was performed by a LMM. *P* < 0.05 are considered as significant: ^*^ *p* < 0.05, ^**^ *p* < 0.01 and ^***^ *p* < 0.001. **a** grey square / grey circle Time point 0 (**§**) vs. 140 (**§§**) min: *p* < 0.001. **d** grey circle Time point 0 (**§**) vs. 140 (**§§**) min: *p* < 0.05

Perfusion of PDGF-BB (final concentration in the buffer: 10 nM) increased P_PA_ up to 116% (*p* < 0.05), whereas P_PA_ remained stable over 140 min in untreated control lungs (Fig. 2a). Pre-treatment with perfused imatinib (final concentration in the buffer: 10 μM) completely prevented the PDGF-BB-related increase of P_PA_ (*p* < 0.05) (Fig. 2a) and even decreased P_PA_ compared to baseline values (*p* < 0.001). Pre-treatment with nebulised imatinib also prevented the PDGF-BB-induced increase of P_PA_ (*p* < 0.05) (Fig. 2a). In addition, the soley perfusion of imatinib significantly decreased P_PA_ compared to baseline values (p < 0.001) (Fig. 2a).

Perfusion of PDGF-BB (final concentration in the buffer: 10 nM) increased P_cap_ up to 193% (p < 0.001) compared to control lungs and to baseline values (Fig. 2b). According to the effects on P_PA_, the PDGF-BB-induced increase of P_cap_ was completely prevented (p < 0.001), if the lungs were pre-treated with perfused or nebulised imatinib (Fig. 2b). Further, perfusion of imatinib lowered P_cap_, at time points 60, 100 or 120 min (for all: 0.04) (Fig. 2b). Neither perfusion of PDGF-BB, nor perfusion of imatinib affected anyhow R_pre_ (Fig. 2c).

Perfusion of PDGF-BB significantly increased R_post_ (*p* < 0.001). This effect was completely prevented, if lungs were pre-treated with imatinib (*p* < 0.001), either perfused or nebulised. Further, perfusion of imatinib alone decreased R_post_ (*p* < 0.05) compared to control lungs and to baseline values (Fig. 2d). The addition of PDGF-BB did not alter P_LA_ (data not shown).

### PCLS: PDGF-BB contracts PAs and PVs via activation of PDGFR-β

**Fig. 3:**
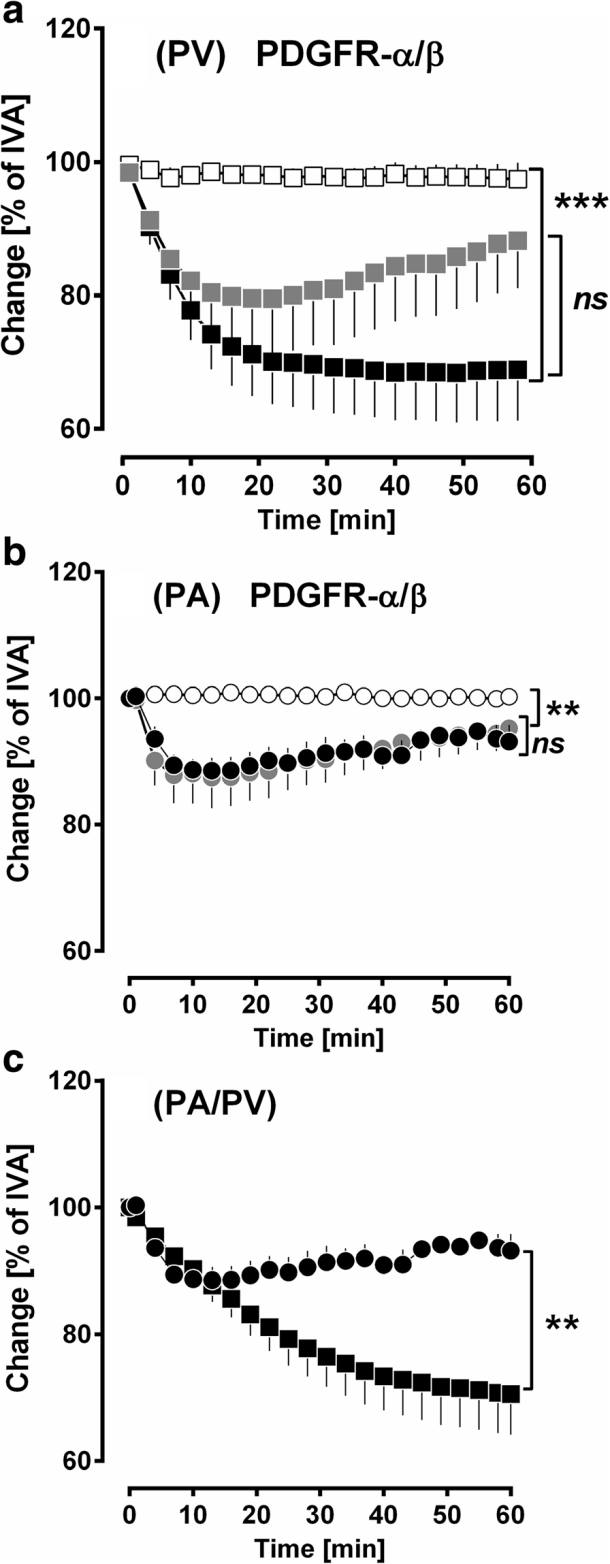
PCLS: PDGF-BB contracts PAs and PVs via activation of PDGFR-β. **a** PDGF-BB contracts PVs: (■) no pre-treatment / 100 nM PDGF-BB (*n* = 7); (grey square) 100 nM ponatinib / 100 nM PDGF-BB (*n* = 7); (□) 5 μM SU6668 / 100 nM PDGF-BB (*n* = 7). **b** The contractile effect of PDGF-BB in PAs: (●) no pre-treatment / 100 nM PDGF-BB (*n* = 7); (grey circle) 100 nM ponatinib / 100 nM PDGF-BB (*n* = 7); (○) 5 μM SU6668 / 100 nM PDGF-BB (*n* = 7). **c** PDGF-BB-induced contraction in PAs/PVs: (●) PAs: 100 nM PDGF-BB (*n* = 7); (■) PVs: 100 nM PDGF-BB (*n* = 7). **a-c** Statistics was performed by a LMM. *P* < 0.05 are considered as significant: ^*^ *p* < 0.05, ^**^ *p* < 0.01 and ^***^ *p* < 0.001

In IPLs, PDGF-BB contracted the pulmonary vascular bed and this was preventable by the TKI imatinib (Fig. 2). Next, we tried to find out in PCLS if PDGF-BB contracts PAs and if this contraction predominantly depends on PDGFR-β, as it was shown for PVs (Fig. 3a) [7]. PDGF-BB contracted PAs up to 87% of IVA (*p* < 0.05) and this contraction was prevented, if PCLS were pre-treated with the PDGFR-β inhibitor SU6668 (*p* < 0.01), whereas pre-treatment with the PDGFR-α inhibitor ponatinib had no effect (Fig. 3b). In PVs, PDGF-BB-induced contraction was stronger than in PAs (*p* < 0.01, Fig. 3c).

### PCLS: Mechanisms beyond PDGF-BB induced contraction in PVs

To get insights if there is a link between PDGF-BB-induced contraction and the pathogenesis of PH, we focused the mechanisms beyond PDGF-BB-induced contraction. Due to the weak contractile effect of PDGF-BB in PAs; we studied PDGF-BB downstream-signalling in PVs.

### The role of calcium in PDGF-BB-induced contraction

**Fig. 4:**
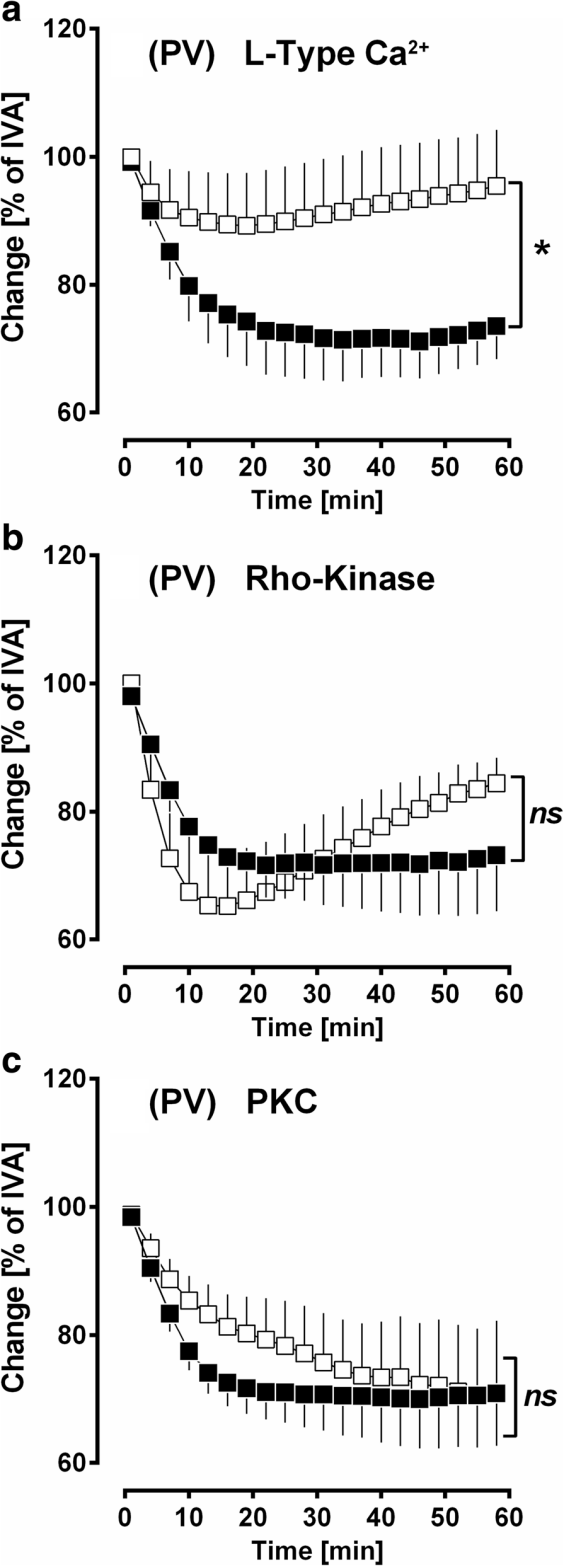
PCLS: The role of Ca^2+^ in PDGF-BB-induced contraction in PVs. **a** PDGF-BB-induced contraction depends on the activation of L-Type Ca^2+^-channels: (■) no pre-treatment / 100 nM PDGF-BB (*n* = 8); (□) 100 nM amlodipine / 100 nM PDGF-BB (n = 8). **b** PDGF-BB-induced contraction does not depend on the activation of Rho Kinase: (■) no pre-treatment / 100 nM PDGF-BB (*n* = 8); (□) 10 μM fasudile / 100 nM PDGF-BB (*n* = 8). **C)** PDGF-BB-induced contraction does not depend on the activation of protein kinase C (PKC): (■) no pre-treatment / 100 nM PDGF-BB (*n* = 8); (□) 5 μM calphostin C / 100 nM PDGF-BB (n = 8). **a-c** Statistics was performed by a LMM. P < 0.05 are considered as significant: ^*^ *p* < 0.05, ^**^ *p* < 0.01 and ^***^ *p* < 0.001

PVs were pre-treated for 60 min with 100 nM amlodipine (L-Type Ca^2+^-channels), 10 μM fasudile (Rho kinase inhibitor) or 5 μM calphostin C (PKC) prior to the application of 100 nM PDGF-BB. Amlodipine significantly reduced the contractile effect of PDGF-BB (p < 0.05) (Fig. 4a), whereas fasudile (Fig. 4b) or calphostin C (Fig. 4c) were without significant effect (*p* > 0.05 for both).

### The role of prostaglandins in PDGF-BB-induced contraction

**Fig. 5:**
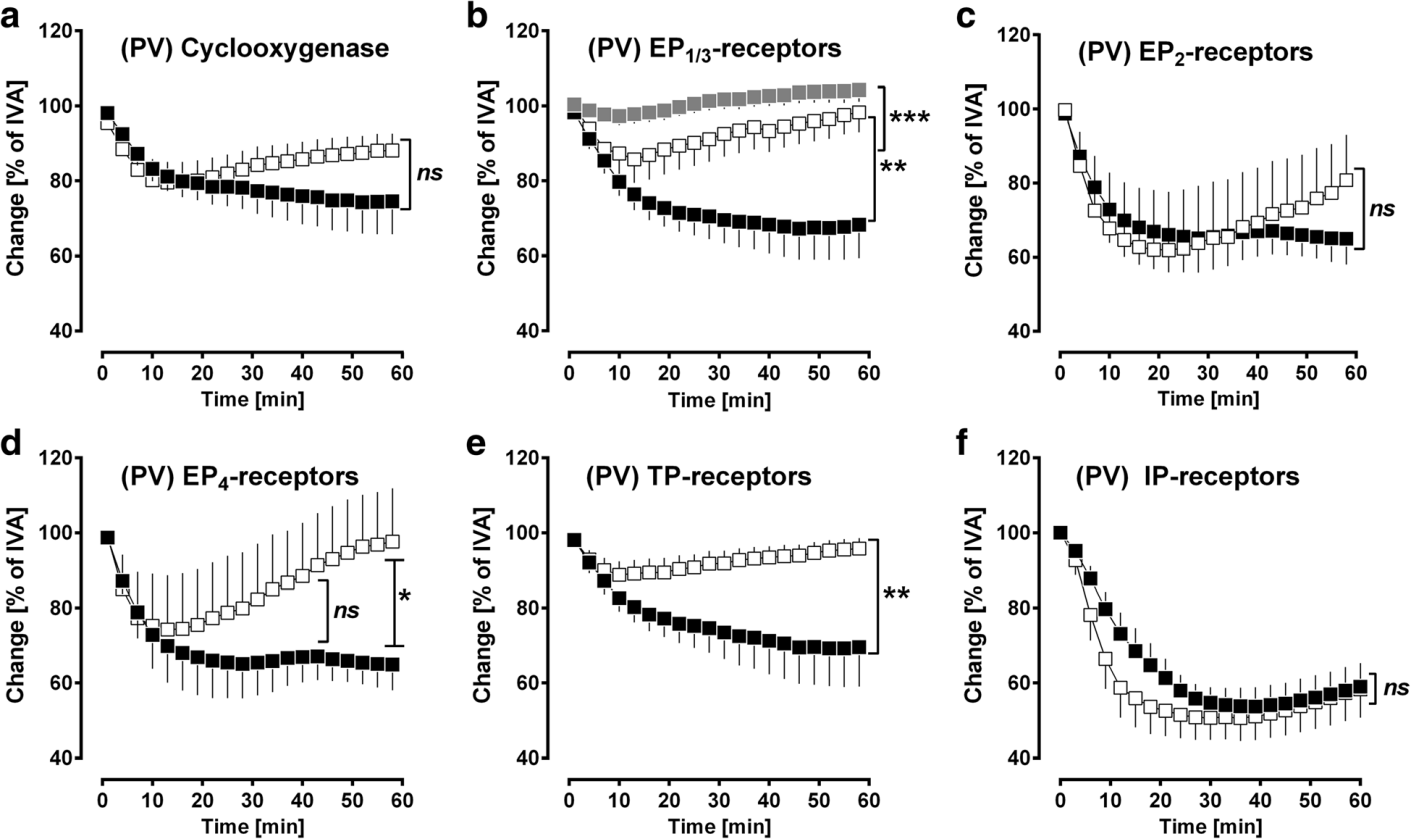
PCLS: The role of prostaglandins in PDGF-BB-induced contraction in PVs. **a** Effect of inhibited cyclooxygenase on PDGF-BB-induced contraction: (■) no pre-treatment / 100 nM PDGF-BB (*n* = 8); (□) 3 μM indomethacin / 100 nM PDGF-BB (*n* = 8). **b** PDGF-BB-induced contraction is mediated via EP_1/3_-receptors: (■) no pre-treatment / 100 nM PDGF-BB (n = 8); (□) 1 μM SC51322 (EP_1_) / 100 nM PDGF-BB (*n* = 8); (grey square) 1 μM L798106 (EP_3_) / 100 nM PDGF-BB (*n* = 7). **c** PDGF-BB-induced contraction does not depend on EP_2_-receptors: (■) no pre-treatment / 100 nM PDGF-BB (*n* = 5); (□) 1 μM PF04418948 / 100 nM PDGF-BB (*n* = 5). **d** PDGF-BB-induced contraction depends on EP_4_-receptors: (■) no pre-treatment / 100 nM PDGF-BB (*n* = 5); (□) 1 μM L161982 / 100 nM PDGF-BB (*n* = 5). **e** PDGF-BB-induced contraction depends on TP-receptors: (■) no pre-treatment / 100 nM PDGF-BB (*n* = 7); (□) 10 μM SQ29548 / 100 nM PDGF-BB (*n* = 7). **f** PDGF-BB-induced contraction does not depend on IP-receptors: (■) no pre-treatment / 100 nM PDGF-BB (*n* = 4); (□) 1 μM RO-1138452 / 100 nM PDGF-BB (*n* = 4). **a-f** Statistics was performed by a LMM. *P* < 0.05 are considered as significant: ^*^ *p* < 0.05, ^**^ *p* < 0.01 or ^***^ *p* < 0.001

Next, we studied, whether the contractile effect of PDGF-BB is mediated via contractile prostaglandins. PCLS were pre-treated with the non-selective cyclooxygenase-inhibitor indomethacin (3 μM), with the EP_1_-receptor antagonist SC51322 (1 μM), with the EP_2_-receptor antagonist PF04418948 (1 μM), with the EP_3_-receptor antagonist L798106 (1 μM), with the EP_4_-receptor antagonist L161982 (1 μM), with the TP-receptor antagonist SQ29548 (10 μM) and with the IP-receptor antagonist RO-1138454 (1 μM). Inhibition of prostaglandin synthesis (indomethacin) did not significantly alter PDGF-BB-induced contraction (Fig. 5a), although the sustained effect of PDGF-BB appeared to be reduced. PDGF-BB-induced contraction was significantly reduced, if EP_1_-receptors (*p* < 0.01; Fig. 5b), EP_3_-receptors (*p* < 0.001; Fig. 5b) or TP-receptors (*p *< 0.01; Fig. 5e) were blocked. In contrast, inhibition of EP_4_-receptors (Fig. 5d) only attenuated PDGF-BB-induced contraction from time point 45 min, whereas inhibition of EP_2_-receptors (Fig. 5c) or IP-receptors (Fig. 5f) did not affect the maximal contractile effect of PDGF-BB. However, pre-treatment with the IP-receptor antagonist RO-1138454 strongly contracted PVs to 75.5% of IVA (*p* < 0.001; data not shown). Finally, inhibition of EP_3_-receptors (Fig. 5b) was most potent and nearly completely prevented PDGF-BB-induced contraction.

**Fig. 6:**
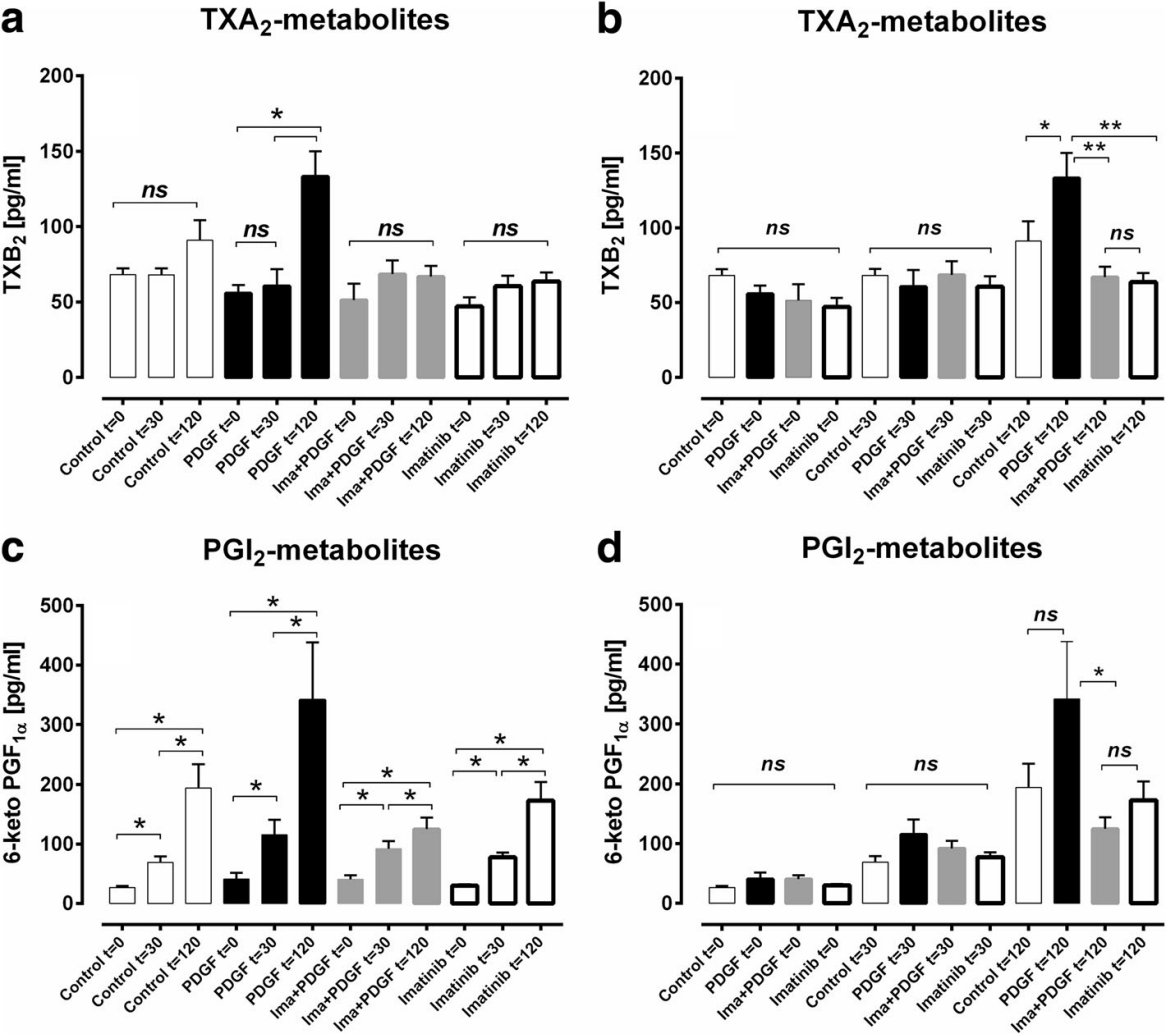
The effect of PDGF-BB on TXB_2_ and 6-keto PGF_1α._ **a** TXB_2_-generation in dependence of the perfusion time. **b** Comparison of TXB_2_-generation within the groups at the same time. **c** 6-keto PGF_1α_-generation in dependence of the perfusion time. **d** Comparison of 6-keto PGF_1α_-generation within the groups at the same time. For all (□) control (*n* = 6); (■) perfusion with PDGF-BB (*n* = 6); (grey square) perfusion with imatinib / PDGF-BB (*n* = 6); (□) perfusion with imatinib (*n* = 6). **a/c** Statistics was performed by the Wilcoxon signed ranked test. **b/d** Statistics was performed by the Mann-Whitney U test. *P* < 0.05 are considered as significant: ^*^ *p* < 0.05 and ^**^ *p* < 0.01

In IPL-perfusate samples, we studied the effect of PDGF-BB on the generation of prostaglandins, e.g. TXB_2_ for TXA_2_ and 6-keto PGF_1α_ for PGI_2_ (Fig. 6). After 120 min of perfusion, PDGF-BB enhanced TXB_2_ compared to basic values (*p* < 0.05; Fig. 6a). Further at 120 min, TXB_2_ was significantly increased compared to 1) the control, 2) the imatinib/PDGF-BB and 3) the imatinib group (p < 0.05; Fig. 6b), whereas at 0 or 30 min, the four treatment groups did not differ (Fig. 6b). In contrast, the PGI_2_-metabolite 6-keto PGF_1α_ was in all groups significantly increased (*p* < 0.05) in dependence to the perfusion time (Fig. 6c). At 120 min, 6-keto PGF_1α_ reached a level of 341 pg/ml in the PDGF-BB group compared to 193 pg/ml in the control group (*p* > 0.05) and to 124 pg/ml in the imatinib/PDGF-BB group (p < 0.05; Fig. 6d). Hence, 6-keto PGF_1α_ was significantly lower, if IPLs were pre-treated with imatinib compared to PDGF-BB alone, although PDGF-BB did not significantly increase 6-keto PGF_1α_ compared to the control group. With regard to 6-keto PGF_1α_, no differences were found at 0 or 30 min (Fig. 6d).

### MAPK-pathway and PI3K-α/γ and AKT/PKB

**Fig. 7:**
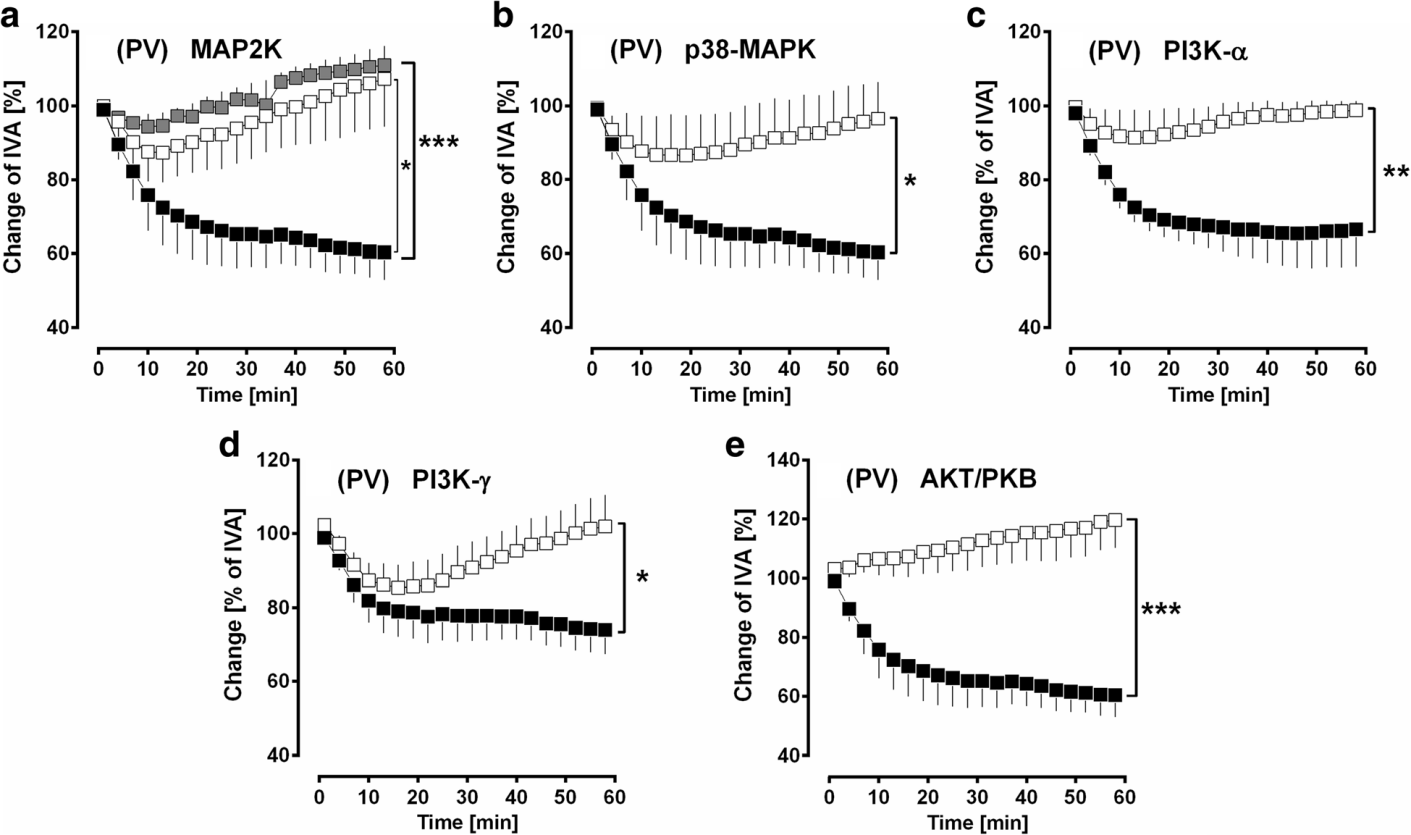
PCLS: PDGFR downstream-signalling: MAPK- and PI3K/AKT/PKB-pathway in PVs. **a** PDGF-BB-induced contraction depends on MAP2K: (■) no pre-treatment / 100 nM PDGF-BB (*n* = 4); (□) 50 μM PD98059 / 100 nM PDGF-BB (*n* = 4); (grey square) 5 μM U0126 / 100 nM PDGF-BB (*n* = 4). **b** PDGF-BB-induced contraction depends on p-38/MAPK: (■) no pre-treatment / 100 nM PDGF-BB (*n* = 4); (□) 5 μM SB203580 / 100 nM PDGF-BB (*n* = 4). **c** PDGF-BB-induced contraction is mediated via PI3K-α: (■) no pre-treatment / 100 nM PDGF-BB (*n* = 6); (□) 1 μM GSK 1059625 / 100 nM PDGF-BB (*n* = 6). **d** PDGF-BB-induced contraction does not interact with PI3K-γ: (■) no pre-treatment / 100 nM PDGF-BB (*n* = 5); (□) 1 μM AS252424 / 100 nM PDGF-BB (*n* = 5). **e** PDGF-BB-induced contraction depends on the AKT/PKB signalling: (■) no pre-treatment / 100 nM PDGF-BB (*n* = 4); (□ 10 μM 10-DEBC / 100 nM PDGF-BB (*n* = 4). **a-e)** Statistics was performed by a LMM. *P* < 0.05 are considered as significant: ^*^ *p* < 0.05, ^**^ *p* < 0.01 and ^***^ *p* < 0.001

Next, we studied if the PDGF-BB downstream-signalling involved in proliferation [3] also contributes to PDGF-BB-induced contraction. Therefore, we inhibited cellular pathways such as MAP2K (5 μM U0126 / 50 μM PD98059), p-38 MAPK (5 μM SB203580), PI3K-α (100 nM GSK 1059625), PI3K-γ (100 nM AS252424) and AKT/PKB (10 μM DEBC). Inhibition of MAP2K (*p* < 0.05, *p* < 0.001; Fig. 7a), p38-MAPK (p < 0.05; Fig. 7b), AKT/PKB (p < 0.001; Fig. 7e), PI3K-α (*p* < 0.5; Fig. 7c) and PI3K-γ (p < 0.05) reduced the contractile effect of PDGF-BB (Fig. 7d).

### PDGF-BB-induced generation of relaxant mediators

**Fig. 8:**
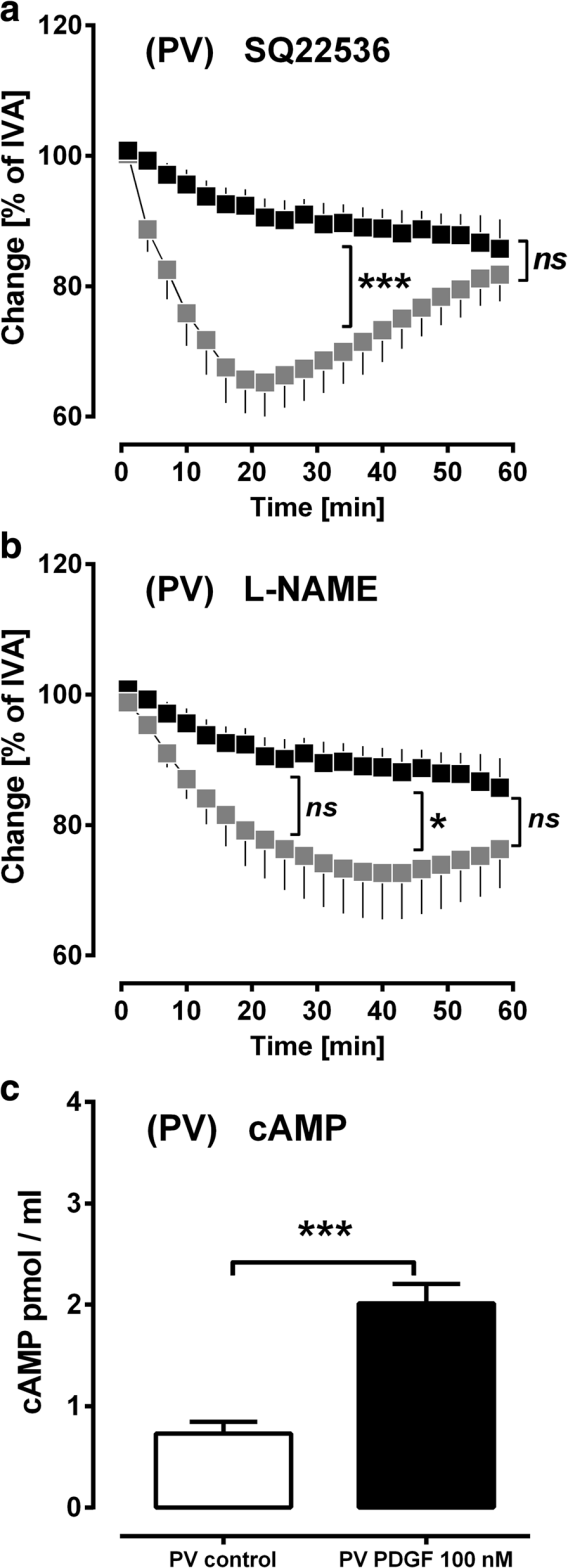
Relevance of relaxant signalling cascades in PDGFR-signalling in PVs. **a** Role of cAMP in PDGFR-signalling: (■) no pre-treatment / 100 nM PDGF-BB (*n* = 10); (grey square) 100 μM SQ22536 / 100 nM PDGF-BB (n = 10). **b** Role of NO in PDGFR-signalling: (■) no pre-treatment / 100 nM PDGF-BB (*n* = 10); (grey square) 100 μM L-NAME / 100 nM PDGF-BB (*n* = 10). **c** PDGF-BB increases intracellular cAMP: (□) no PDGF-BB (control) (*n* = 10); (■) 100 nM PDGF-BB (*n* = 11) **a/b** Statistics was performed by a LMM. **C)** Statistics was performed by the Mann-Whitney U test. *P* < 0.05 are considered as significant: ^*^ *p* < 0.05, ^**^ *p* < 0.01 and ^***^ *p* < 0.001

The data from Fig. 7a/e reveal weak relaxation due to PDGF-BB leading to an IVA > 100%. Together with the observations that some PVs contract to less than 80% of IVA (Fig. 7a; *p* = 0.003), the idea was obvious that PDGF-BB downstream-signalling might involve relaxant mediators different from PGI_2_ (Fig. 6c/d), e.g. cAMP or NO. To study this issue, PVs were pre-treated with the AC-inhibitor SQ22536 or with the endothelial NOS-inhibitor L-NAME prior to the application of PDGF-BB. Inhibition of cAMP-generation (SQ22536) increased the contractile effect of PDGF-BB until time point 35 min (p < 0.001) (Fig. 8a), whereas inhibition of endothelial NOS (eNOS) only slightly enhanced it (Fig. 8b), as this increase only reached statistical significance between the time points 30–45 min (*p* < 0.05; Fig. 8b). Further, 100 nM PDGF-BB increased cAMP (*p* < 0.001) in PVs (Fig. 8c) suggesting that PDGF-BB-induced generation of cAMP influences the pulmonary venous tone, whereas PDGF-BB-related NO-synthesis appears to play a minor role in PVs.

### The role of actin polymerisation in PDGF-BB-induced contraction

**Fig. 9:**
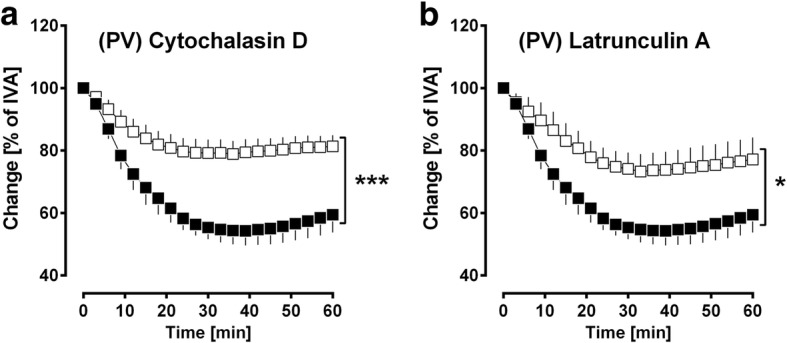
The role of actin polymerisation in PDGF-BB-induced contraction. **a**. Inhibition of actin polymerisation by cytochalasin D: (■) no pre-treatment / 100 nM PDGF-B (*n* = 4); (□) 10 μM cytochalasin D / 100 nM PDGF-BB (*n* = 4). **b** Inhibition of actin polymerisation by latrunculin A: (■) no pre-treatment / 100 nM PDGF-BB (*n* = 4); (□) 1 μM latrunculin A / 100 nM PDGF-BB (*n* = 4). **a/b** Statistics was performed by a LMM. *P* < 0.05 are considered as significant: ^*^ *p* < 0.05 and ^***^ *p* < 0.001

PDGF-BB-induced contraction appears to depend on complex intracellular pathways. Next, we analysed the role of actin polymerisation by 10 μM cytochalasin D or 1 μM latrunculin A. Inhibition of actin polymerisation significantly lowered the contractile effect of PDGF-BB, as indicated for cytochalasin D (*p* < 0.001; Fig. 9a) and latrunculin A (*p* < 0.05; Fig. 9b).

## Discussion

PDGF and PDGFR play a critical role within the remodelling in PH [1, 2]. We show that PDGF-BB contracts the pulmonary vascular bed of GPs via activation of PDGFR-β. In PVs, PDGF-BB-induced contraction depends on L-Type Ca^2+^-channels, PI3K-α/γ, MAPK- and AKT/PKB-signalling and actin remodelling. Beyond that, stimulation of EP_1/3_- or TP-receptors plays a significant role in PDGF-BB-induced contraction, whereas stimulation of IP-receptors is not relevant. In addition, PVs treated with PDGF-BB show increased cAMP levels which do not appear to rely on PGI_2_.

### Effects of PDGF-BB on the pulmonary vascular bed

In the IPL, recirculating perfusion of 10 nM PDGF-BB significantly enhanced P_PA_ up to 116% (Fig. 2a). These results confirmed those from PCLS; where 100 nM PDGF-BB contracted PAs up to 87% of IVA (Fig. 3b). According to the Hagen-Poiseuille law, the resistance increases 16 fold, if the radius is splitted in half. Hence, P_PA_ would have increased even above 116%, if lungs were perfused with 100 nM PDGF-BB. Further in the IPL, PDGF-BB-induced alteration of the vascular tone is detected even at lower concentrations compared to PCLS. This is supported by the fact that 10 nM PDGF-BB did not contract GPs’ PAs in PCLS (data not shown), whereas 10 nM PDGF-BB enhanced P_PA_ to 116% in the IPL (Fig. 2a). In contrast to these results, PDGF-BB did not alter R_pre_ (Fig. 2c) indicating a minor effect on the cavine precapillary pulmonary vascular bed. Possible reasons for this observation might be 1) a lower receptor density; 2) a varying receptor equipment with reduced sensitivity. Most probably, our results regarding the effect of PDGF-BB on R_pre_ are not transferable to the human situation, as small human PAs are equipped with PDGFR-β [1]. In general, GPs’ PCLS allow to study more central pulmonary vessel, but do not represent the precapillary part of the pulmonary circulation [13]. In contrast, the IPL allows addressing the entire pulmonary vascular bed (except central PVs); particularly, it enables to determine the segmental vascular resistance (R_pre_ / R_post_) by the double occlusion method [7, 13, 14]. Beyond PDGF-BB-induced pulmonary arterial contraction, PDGF-BB increased P_cap_ (Fig. 2b) and R_post_ (Fig. 2d) up to 200 and 140% of baseline values, respectively. Further, PDGF-BB contracted central PVs from GPs up to 70% (Fig. 3a).

Hence, our GP’ data from both models suggest that PDGF-BB exerts significant contraction along the pulmonary vascular bed and give strong evidence that PDGF-BB-induced contraction is accentuated in the pulmonary venous system PVs (Fig. 2; Fig. 3). This result is of high clinical relevance, as the pulmonary venous bed contributes about 40% to pulmonary vascular resistance (PVR) [60] and plays a major role in PH due to left heart disease [61], the most common cause of PH [62, 63].

### PDGF-BB contracts the pulmonary vascular bed via activation of PDGFR-β

PDGF-BB-induced contraction of the pulmonary vascular bed was completely prevented if IPLs (Fig. 2) were pre-treated with the PDGFR-α/β inhibitor imatinib, either perfused or nebulised; indicating that PDGF-BB-induced contraction specifically relies on the activation of PDGFR-α/β. With regard to GPs’ PVs, we recently showed that PDGF-BB-induced contraction mainly depends on the activation of PDGFR-β (Fig. 3a), whereas activation of PDGFR-α only plays a minor role [7]. In this work, we validated this also for GPs’ PAs as we found that inhibition of PDGFR-β (SU6668) completely prevented PDGF-BB-induced contraction of PAs (Fig. 3b) and inhibition of PDGFR-α (ponatinib) had no effect (Fig. 3b). In the IPL, perfusion of imatinib did not only prevent the PDGF-BB-induced increase of P_PA_, P_cap_ and R_post_, but also decreased P_PA_, R_post_ and in part P_cap_ (time points 90 and 110 min after addition) compared to untreated control lungs. These data suggest the existence of endogenously produced PDGF-BB and the permanent activation of PDGFR.

### Mechanisms beyond PDGF-BB induced contraction

After identification of PDGF in the seventies, PDGF-BB-induced contraction [8–10, 64] and relaxation [11, 12] was proven in systemic arteries. Afterwards, the vascular effects of PDGF disappeared in the background and research focused on the proliferative effects of PDGF [1, 2] leading to the introduction of TKIs in the therapy of PH [2, 4, 65]. With this regard, TKI-induced relaxation has been uncovered [5–7] and the contractile effects of PDGF-BB have been proven in PVs [7]. Thus, it becomes apparent that PDGF-BB promotes aside proliferation also contraction of PAs/PVs, both promoting the progress of PH. Therefore we studied the mechanisms beyond the contractile effect of PDGF-BB in GPs’ PVs.

### The role of calcium in PDGF-BB-induced contraction

In PVs, PDGF-BB-induced contraction depended on the activation of L-Type Ca^2+^-channels (Fig. 4a), whereas Ca^2+^-sensitisation did not play a role, as inhibition of Rho-Kinase or PKC did not alter PDGF-BB-induced contraction (Fig. 4b/c). In line with our results, PDGF-AB or PDGF-BB contract extra pulmonary vessels in a calcium dependent manner [8–10, 64, 66] and vice versa, TKIs modulate the activity of L-Type Ca^2+^-channels in portal veins [67, 68]. Anyhow, differences exist within the PDGF dimers AA, AB or BB; e.g. Sachinidis et al. [10] reported in rats’ aortic rings that PDGF-BB contracts stronger and rises intracellular calcium more potently than PDGF-AB, whereas PDGF-AA acts only poorly contractile and does not rise intracellular calcium [10]. In rat aortic smooth muscle cells (SMCs), the same group [10, 69] found that PDGF-AA potently stimulates PKC, whereas PDGF-BB activates PKC only in a minor degree. These findings suggest that PDGF-AA rather acts via Ca^2+^-sensitisation, whereas PDGF-BB mainly exerts contraction via the increase of calcium. Moreover, with regard to tone or endothelial barrier, pulmonary and systemic vessels are diversely regulated [70]. This circumstance might also explain contrasting results.

### The role of prostaglandins in PDGF-BB-induced contraction

So far, it is unknown, if prostaglandins mediate the contractile effect of PDGF-BB in pulmonary vessel. Although, PDGFR downstream-signalling is linked to the generation of prostaglandins [71–73] and prostaglandin receptors, e.g. TP- or EP_1/3_-receptors are involved within the regulation of the tone of human PAs [74] and PVs [75, 76].

Now, our data reveal that PDGF-BB-induced contraction goes ahead with the activation of TP-receptors, as 1) inhibition of TP-receptors strongly reduced the contractile effect of PDGF-BB and as 2) TXB_2,_ the inactive metabolite of TXA_2_ was significantly enhanced in PDGF-BB perfused lungs. TXA_2_ acts as a potent vasoconstrictor and highly contributes to increased vascular tone in PH [77–79]. TP-receptors represent G-Protein-coupled receptors (GPCR) which are mainly coupled to G_αq/11_ and G_α12/13_, but also to G_αs/i_, G_h_ and G_βγ_; finally their stimulation leads to the regulation of phospholipase C (PLC)/inositol trisphosphate (IP_3_)/calcium, Rho and AC [80]. Beyond that, MAPK- and PI3K-signalling is also involved [80]. In line with our data, Sachinidis et al. [10] showed in systemic vessels that PDGF-BB leads to the generation of TXA_2_ which exerts as well as the TP-agonist U46619 a strong and long-lasting contraction along the pulmonary vascular bed [81–84]. Our data show no involvement of Rho/PKC in PDGF-BB downstream-signalling. This is opposing to Murtha et al. [84] who showed in rabbits’ pulmonary arterial rings that the contractile effect of TXA_2_ depends on PKC. Further, TP-receptors are coupled to G_α12/13_, hence their stimulation should activate Rho [85], unless TP-receptors are primarily coupled to G_αq/11_ [80]. Notably, vasoconstrictors such as endothelin-1 or platelet-activating factor also mediate their contractile effect via the release of TXA_2_ [86, 87].

Our results indicate that PDGF-BB-induced contraction goes ahead with the activation of EP_1/3/4_-receptors, whereas stimulation of EP_2_-receptors does not play a role (Fig. 5c). Hence, PGE_2_ as the most widely produced prostaglandin of the body binding to EP_1–4_-receptors (GPCRs) [25] appears to be highly involved in PDGF-BB-induced contraction. EP_1_-receptors are coupled to G_αq/11_ [25, 88] and their activation triggers the intracellular increase of PLC, IP_3_ and calcium [88]. In contrast, EP_3_-receptors are mainly coupled to G_αi_ and their activation inhibits the AC leading to decreased cAMP-levels [25, 88]. Here, inhibition of EP_3_-receptors nearly completely prevented PDGF-BB-induced contraction, although inhibition of EP_1_- or TP-receptors was also very effective in preventing the contractile effect of PDGF-BB. So, the question comes up if the EP_3_-receptor antagonist L798106 acts unspecific and also binds to EP_1_- or TP-receptors. According to IC_50_ values, this is not the case [48]. However, the prominent effect of EP_3_-inhibition on PDGF-BB-induced contraction might be explainable by the consideration that EP_3_-inhibition may provoke an overwhelming cAMP-generation counteracting other contractile mediators activated by PDGF-BB. In general, EP_3_-agonists strongly contract human PAs [74]. Beyond that, they influence the progress of PH, as EP_3_-receptor deficiency attenuates the expression of PH [89]. Aside EP_1/3_-receptors, PGE_2_ also activates EP_2_- and EP_4_-receptors which are both coupled to G_αs_ leading to the activation of AC, to the increase of cAMP and to reduced vessel tone [25, 88]. Moreover, EP_4_-receptors also couple to G_αi_ representing the counter player of G_αs_ [25, 88]. Here, inhibition of EP_2_-receptors did not enhance the contractile effect of PDGF-BB, inhibition of EP_4_-receptors reduced PDGF-BB-induced contraction and PVs treated with PDGF-BB showed increased cAMP-levels. These data suggest 1) a possible minor expression of EP_2_-receptors in GPs’ PVs or 2) a possible dominant coupling of EP_4_-receptors to G_αi_ [90] which has been already reported in the hypoxic pulmonary arterial bed of the rat. In addition, although EP_4_-receptors appear to couple dominantly to G_αi_, G_αs_-coupling seems to be of relevance, as PDGF-BB significantly increased cAMP-levels in PVs. This idea is supported by the circumstance that PGI_2_, as a main source for cAMP [91] did not significantly increase due to PDGF-BB. Thus, the production of cAMP should derive from PDGF-BB-induced activation of prostaglandin receptors others than IP, but also coupled to G_αs_.

In general, the various prostaglandins mediate contraction or relaxation. In this work, EP_1/3/4_- and TP-receptors reduced the contractile effect of PDGF-BB, whereas none of the inhibitors, including the IP-receptor antagonist RO-1138452, did increase PDGF-BB-induced contraction. At first glance, PDGF-BB-downstream-signalling appears to be dominated by the generation of contractile prostaglandins. At second view, this assumption is opposed, as inhibition of prostaglandin synthesis (indomethacin) did not significantly alter PDGF-BB-induced contraction (Fig. 5a); a fact which suggests that PDGF-BB-dependent prostaglandin generation is well-balanced between contractile and relaxant ones. Further, inhibition of MAP2K- and AKT/PKB-signalling (Fig. 7a/e) unmasked a slight relaxant effect of PDGF-BB supporting the hypothesis that PDGFR-downstream-signalling is anyhow related to relaxant pathways. Our results indicate that PGI_2_ is of less relevance within the regulation of the pulmonary vascular tone by PDGF-BB, as 1) inhibition of IP-receptors did not enhance the contractile effect of PDGF-BB (Fig. 5f). 2) PDGF-BB did not increase PGI_2_ (Fig. 6c/d), though a trend appears to be evident which is enforced by the fact that pre-treatment with imatinib significantly lowered PGI_2_-levels compared to PDGF-BB perfusion alone. The observation that PGI_2_ increased time-dependently in the perfusate of all IPL-groups (Fig. 6c), including control lungs is explainable by the endothelial release of PGI_2_ counteracting the increased shear stress in perfused lungs [92, 93]. In general, the release of PGI_2_ strongly depends on shear stress [94]. In contrast, in PCLS shear stress is hardly effective [95], hence time-dependent PGI_2_-release is not expected. However, we could show that basal PGI_2_-release should occur, as inhibition of IP-receptors increased the tone of PVs. Finally, our data indicate that PDGF-BB-induced PGI_2_-release is less relevant, although we cannot exclude that PDGF-BB potentiates anyhow the release of PGI_2_ due to shear stress. Our results are different from those of Yamawaki et al. [12] who proved that PDGF-BB relaxes rat mesenteric arteries in dependence to the release of PGI_2_. Finally, the role of PDGF-BB-induced PGI_2_-release might depend on the vessel localisation, e.g. pulmonary vessels versus systemic vessels and on the species.

In spite of the fact that PDGF-BB contracts GPs’ PVs, the generation of relaxant mediators such as cAMP and cGMP plays a relevant role in PDGFR-downstream-signalling. 1) Inhibition of AC (Fig. 8a) enforced the contractile effect of PDGF-BB, 2) PVs treated with PDGF-BB had higher cAMP-levels than control PVs (Fig. 8c) and 3) inhibition of eNOS slightly enforced the contractile effect of PDGF-BB (Fig. 8b). Our results are supported by those of Graves et al. [96] who found in human arterial SMCs that PDGF-BB downstream-signalling goes ahead with the generation of cAMP/PKA, just as the cAMP generation depends on the release of arachidonic acid, probably activating EP_2/4_- or IP-receptors. These results were also proven in rat myometrial cells [71] and in GPs’ airway SMCs [97]. Usually, stimuli which activate prostaglandin receptors coupling to G_αs_; e.g. EP_2/4_, IP or DP should increase intracellular cAMP [98, 99].

Aside from cAMP, NO seems to be of impact in PDGF-BB downstream-signalling. Though, NO-inhibition only slightly enhanced PDGF-BB-induced contraction (Fig. 8b). Our results are in line with those from Takase et al. [11] who perfused rat mesenteric arteries; there PDGF-BB stimulated NO-release even relaxed rat mesenteric arteries. The different characteristic of PDGF-BB-induced NO-release might be due to two facts; 1) Takase et al. [11] exposed rat mesenteric arteries to shear stress, generally going ahead with endothelial NO-release [100], 2) systemic and pulmonary vessel behave different to similar stimuli [70].

### PDGFR downstream-signalling: MAPK-pathway and PI3K-α/γ and AKT/PKB

With regard to cellular regulation (migration, differentiation, proliferation, growth or survival of cells), PDGFR downstream-signalling mainly activates two pathways: 1) the MAPK-pathway and 2) the PI3K/AKT/mTOR pathway [101]. Our data show that both inhibition of MAP2K by PD98059 or U-0126, as well as inhibition of p38-MAPK by SB 203580 almost prevented the contraction by PDGF-BB (Fig. 7a/b). Notably, GPs’ PVs even relaxed slightly (Fig. 7a). In line with our data, Schaafsma et al. [102] showed in GPs’ tracheal strips that PDGF-BB-induced contraction highly depends on the activation of MAP2K. Next, Boulven et al. [71] proved in rat myometrial cells that PDGF-BB-dependent synthesis of prostaglandins is up to MAP2K. Conversely, the stimulating effect of PDGF-BB on phospholipase A_2_ (PLA_2_) and subsequent prostaglandin synthesis also depends on MAP2K [71, 103–105]. Finally, MAPK-signalling is highly involved in PDGF-BB-induced prostaglandin synthesis.

The PI3K/AKT/mTOR pathway highly contributes to mediate the proliferative aspects of PDGFR [3, 101]. Here both, PI3K-α which is expressed ubiquitously [106] and PI3K-γ which is expressed in the cardiovascular system [106] contribute to the contractile effect of PDGF-BB. Hence, PI3K-γ does not only regulate the systemic vascular tone [106], but is also of impact for the regulation of the pulmonary vascular tone. Together with the fact that inhibition of AKT/PKB (Fig. 7e) prevented the contractile effects in PVs, our results suggest a role of PI3K/AKT/mTOR-signalling within the contractile effect of PDGF-BB. Our data are supported by Hua et al. [107] who showed that AKT prevents the degradation of cytosolic PLA_2_ (cPLA_2_), finally promoting prostaglandin synthesis. Beyond that, activation of AKT is linked to the activation of eNOS [108], an issue which seems to be negligible within PDGF-BB-induced regulation of the pulmonary venous tone, as 1) inhibition of AKT did not contract PVs and 2) as inhibition of eNOS enhanced the contractile effect of PDGF-BB only slightly (Fig. 8b). Further, our results are contrasting to those of Macrez et al. [109] who showed in vascular SMCs that the PDGF-BB-induced intracellular increase of calcium depends on PI3K-β, but not on PI3K-α which are both coupled to receptor tyrosine kinases (RTKs) [110–112]. Regarding PDGF-BB-signalling, non-direct activation of MAPK and PI3K is conceivable, as TP-receptors are also linked to G_βγ_, finally leading to the activation of MAPK- or PI3K/Akt/mTOR signalling [80].

**Fig. 10:**
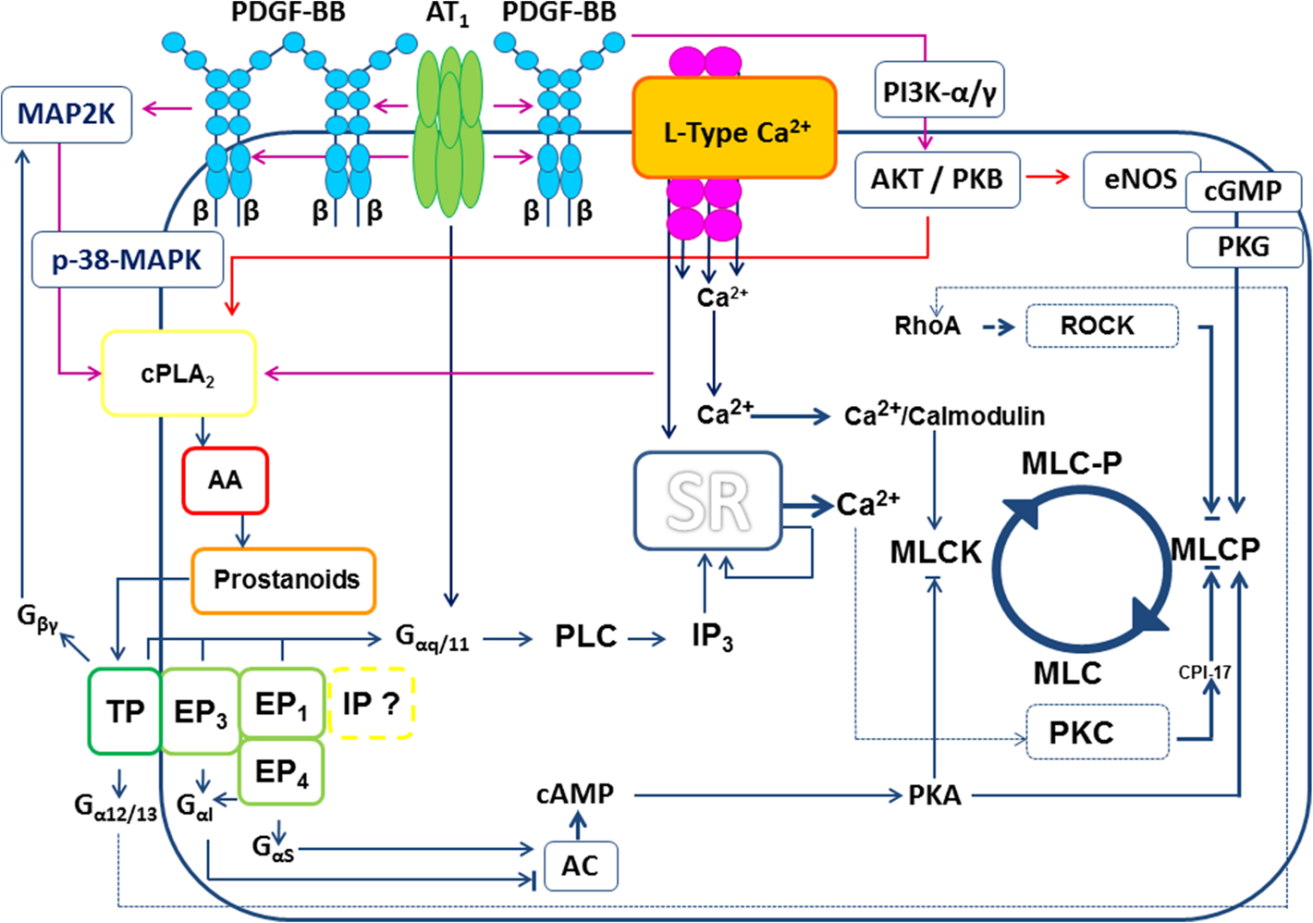
Complex involvement of prostaglandins within the contractile effect of PDGF-BB. The contractile effect of PDGF-BB depends on the activation of L-Type Ca^2+^-channels and on calcium [9, 10, 64], promoting the activation of myosin light chain kinase (MLCK) and vascular SMC contraction [24]. Increased intracellular calcium-levels activate cPLA_2_ [19] leading to the formation of arachidonic acid (AA) [131]. Cytosolic PLA_2_ is also activated by MAP2K−/p-38-MAPK-signalling [71, 103], whereas PI3K- or AKT-signalling prevent its degradation [107]. AA serves as a substrate for the production of prostaglandins e.g. PGE_2_ or TXA_2_ [132] which bind to EP- or TP-receptors. EP_1/3_- or TP-receptors are mainly coupled to G_αq/11_, leading to the formation of IP_3_ and to the release of calcium from the sarcoplasmic reticulum (SR) [25, 88]. TP-receptors are also linked to G_βγ_ activating MAPK-signalling [80]. Further, TP-receptors are coupled to G_α12/13_ [25, 88] activating Rho/ROCK and inhibiting myosin light chain phosphatase (MLCP) [24]. Vascular SMC contraction is further enhanced, if PKC inhibits MLCP [24]. Beyond G_αq/11,_ EP_3_-receptors are coupled to G_αi_ inhibiting cAMP-generation, whereas EP_4_-receptors are coupled to G_αs_, promoting cAMP-generation [25]. Cyclic AMP induces the formation of protein kinase A (PKA) which inhibits MLCK [23] and activates MLCP [133]. The role of PGI_2_ and IP-receptor-activation is less relevant within PDGFR-downstream signalling, but is not completely solved. PDGFR-signalling also activates AKT/PKB [101] which stimulates eNOS [108]. Last, transactivation of AT_1_-receptors and PDGFR [101] is described leading to the increase of calcium [134]. The dashed line indicates pathways which are in contrast to our results

In conclusion, prostaglandin generation appears to be a major mechanism beyond PDGF-BB-induced regulation of the pulmonary venous tone. Within this context, there are several possibilities to activate cPLA_2_, 1) by the increase of intracellular calcium [71, 104, 113], 2) by PDGF-BB-induced MAPK-signalling [71, 103–105] and 3) by the inhibitory properties of AKT on cPLA_2_-degradation [107]. In addition, 4) the transactivation of PDGFR by the G_αq_-coupled AngII is described [101], leading to the activation of PLC and IP_3_ and to the subsequent increase of intracellular calcium. For an overview, please see Fig. 10.

### The role of actin polymerisation in PDGF-BB-induced contraction

Aside MLC-phosphorylation, actin polymerisation plays an important role within the contractile process of SMCs [26, 27]. Within this context, it is of interest that PDGF-BB – via activation of SRC - stimulates the abelson tyrosine kinase (ABL) [114–116] which itself promotes actin polymerisation [27]. In contrast, the TKI imatinib is known to inhibit ABL [27].

Our data indicate that PDGF-BB-induced actin polymerisation contributes to the contractile effect of PDGF-BB, as inhibition of actin polymerisation by cytochalasin D (Fig. 9a) or latrunculin A (Fig. 9b) strongly reduced PDGF-BB-induced contraction in PVs. With regard to the stimulating effect of PDGF-BB on ABL, our results are comprehensible. They are even less unexpected, as MAPK-signalling which represents a cornerstone of PDGF-BB downstream signalling influences actin polymerisation [117, 118]. So far, the impact of actin polymerisation for the regulation of the pulmonary venous tone has not been shown. Although, its relevance for SMC-contraction was shown in SMCs of various vessel and species; e.g. canine carotids [119], ferret aorta [120], rat mesenteric arteries [121, 122], rat thoracic aorta [57] or rat extrapulmonary PAs [123], as well as in SMCs from airways [124].

### Link between PDGF-BB induced pulmonary vascular contraction and remodelling in PH

PDGFR-downstream-signalling is associated with the increase of intracellular calcium [9, 10, 64]. Here we show that the contractile effect of PDGF-BB also depends on it. Increased calcium-levels represent a major trigger for vasoconstriction, proliferation and migration of vascular SMCs [18, 80, 125–127]. Thus, stimuli which increase calcium-levels, e.g. TXA_2_ or ET-1 [80, 128], but also hypoxia [125, 127] or PDGF-BB [2, 64] do not constrain to increase the pulmonary vascular tone, but also promote pulmonary vascular remodelling. Hence, enhanced tone and remodelling are closely linked with each other. In PH, this circumstance could be beneficial in view of new therapeutics, e.g. therapeutics addressing the mechanisms beyond PDGF-BB-induced contraction may attenuate vasoconstriction and remodelling. Ultimately, calcium-levels could be adressed directly by amlodipine or nifedipine, but also indirectly via inhibition of TP/EP_1/3_- receptors. In respect thereof it is worth mentioning that EP_3_-receptor deficiency attenuates PH [89] and that prednisolone inhibits PDGF-BB-induced proliferation of PAs’ SMCs [129]. Further, inhibition of MAP2K-/AKT-signalling and cPLA_2_ could be of interest. Notably, the mentioned pathways could be addressed in a systemic way, but also topically, e.g. via inhalation to reduce systemic side effects.

## Conclusions

PDGF-BB contracts pulmonary vessels. The PDGF-BB related pulmonary vascular effects are prevented by the TKI imatinib (perfused or nebulised). The mechanisms beyond PDGF-BB-induced contraction depend on actin polymerisation, the intracellular increase of calcium, activation of EP_1/3/4_- and TP-receptors and MAP2K- or PI3K/AKT-signalling. In addition, PDGF-BB induces the release of TXA_2_ and cAMP. Finally, aside the known proliferative effects of PDGF-BB in PH, PDGF-BB-induced contraction might also contribute to the pathogenesis of PH. Thus, TKI-inhibtion appears to be beneficial and particularly nebulised imatinib might prevent systemic side effect [130].

## Abbreviations

6-keto PGF_1α_: 6-keto prostaglandin F_1α_
AA: Arachidonic acid
ABL: Abelson tyrosine kinase
AC: Adenyl cyclase
Cdyn: Dynamic compliance
cPLA_2_: Cytosolic phospholipase A_2_
eNOS: Endothelial NOS
EP-receptor: Prostaglandin E_2_ receptor
GPCR: G-Protein-coupled receptor
GPs: Guinea pigs
IP_3_: Inositol trisphosphate
IPL: Isolated perfused lung
IP-receptor: Prostacyclin receptor
IVA: Initial vessel area
LMM: Linear mixed model analysis
MLCK: Myosin light chain kinase
MLCP: Myosin light chain phosphatase
NOS: NO-synthase
PAs: Pulmonary arteries
P_cap_: Capillary pressure
PCLS: Precision-cut lung slices
PDGF: Platelet derived growth factor
PDGFR: PDGF-receptor
PGE_2_: Prostaglandin E_2_
PGI_2_: Prostacyclin (prostaglandin I_2_)
PH: Pulmonary hypertension
PKA: Protein kinase A
PKC: Protein kinase C
P_LA_: Left atrial pressure
PLA_2_: Phospholipase A_2_
PLC: Phospholipase C
P_PA_: Pulmonal arterial pressure
PVR: Pulmonary vascular resistance
PVs: Pulmonary veins
Res: Resistance
R_post_: Postcapillary pressure
R_pre_: Precapillary pressure
RTK: Receptor tyrosine kinase
SMC: Smooth muscle cell
SR: Sarcoplasmic reticulum
TKIs: Tyrosine kinase inhibitors
TP-receptor: Thromboxane prostanoid receptor
TV: Tidal volume
TXA_2_: Thromboxane A_2_
TXB_2_: 11-dehydro TXB_2_ and 2,3-dinor
